# Comparative Assessment of Functional Components and Antioxidant Activities between *Hippophae rhamnoides* ssp. *sinensis* and *H. tibetana* Berries in Qinghai–Tibet Plateau

**DOI:** 10.3390/foods12020341

**Published:** 2023-01-11

**Authors:** Tingting Su, Juan Wei, Jinmei Zhao, Yumei Jiang, Yang Bi, Galitsyn George

**Affiliations:** 1 College of Food Science and Engineering, Gansu Agricultural University, Lanzhou 730070, China; 2 The Federal Research Center, Institute of Cytology and Genetics, Siberian Branch of the Russian Academy of Sciences, 630090 Novosibirsk, Russia

**Keywords:** sea buckthorn berry, soluble sugars, organic acids, antioxidant components, antioxidant activity

## Abstract

The Qinghai–Tibet Plateau is the main production area of *Hippophae rhamnoides* ssp. *sinensis* (Rha) and *H. tibetana* (Tib), but studies on the types and contents of soluble sugars, organic acids, free phenolics, bound phenolics, vitamin C (V_C_), tocopherol (V_E_) and carotenoids of the two sea buckthorn berries from this region have not been reported. In this paper, we found that the soluble sugars in Rha and Tib were mainly glucose and fructose; Rha exhibited a higher content of total sugar and fructose compared to Tib. The organic acids were mainly quinic acid and malic acid; Rha exhibited a higher content of total acids and quinic acid, but lower tartaric acid and citric acid compared to Tib. Rha also possessed a higher total (free and bound) phenolic as well as total (free and bound) flavonoid content than those in Tib; twelve phenolic compounds were analyzed, among which flavonols were dominant. Catechin, isorhamnetin and quercetin were the main phenolic substances. V_C_ and V_E_ (*γ*-tocopherol (*γ*-V_E_) and *δ*-tocopherol (*δ*-V_E_)) were higher in Rha than Tib. The total carotenoid, lutein, *β*-carotene and lycopene content of Tib was remarkably higher than that in Rha. Moreover, both Rha and Tib showed good in vitro and cellular antioxidant activities, and Rha had a stronger antioxidant activity. Taken together, Rha had a higher antioxidant activity, which may be due to its higher content of phenolics, flavonoids, V_C_ and V_E_.

## 1. Introduction

China is rich in sea buckthorn resources, accounting for over 90% of the world’s total production [1]. *H. rhamnoides* subsp. *sinensis* (Rha) is mainly distributed on the Inner Mongolia Plateau, the Loess Plateau and the Qinghai–Tibet Plateau, accounting for approximately 85% of all sea buckthorn in China. *H. tibetana* (Tib) is a sea buckthorn endemic to the Qinghai–Tibet Plateau [2]. Sea buckthorn berries are rich in bioactive components such as phenolics, flavonoids, vitamins and carotenoids, which have a variety of benefits to human health [3,4], such as their antioxidant and anti-inflammatory effects.

Large differences in soluble sugars, organic acids, antioxidant components and antioxidant activities have been reported among sea buckthorn. It is reported that the soluble sugars of sea buckthorn are dominated by glucose and fructose, and organic acids include quinic acid, malic acid, citric acid and oxalic acid [5]. The total phenolic and total flavonoid content of the ethanolic extract of Rha were significantly higher than those of Tib [6]. The vitamin C (V_C_) content of Rha was 5-to 10-fold higher than that in *Hippophae rhamnoides* L. and *H. rhamnoides* subsp. *Mongolica* [7]. Tocopherol (V_E_) content in Rha was remarkably higher than that in *H. rhamnoides* subsp. *Mongolica* [8]. Previous examination has revealed that sea buckthorn berries contain 41 types of carotenoids, of which *β*-carotene, zeaxanthin and *β*-cryptoxanthin were dominant [9]. It has also been reported that the five varieties of sea buckthorn from Poland are mainly all-*trans*-*β*-carotene and *β*-cryptoxanthin [10]. Furthermore, the oxygen radical absorbance capacity, peroxyl radical scavenging capacity and cellular antioxidant activity of Rha were observably higher than other subspecies [11]. Meanwhile, Rha showed significantly higher 2,2-diphenyl-1-picrylhydrazyl (DPPH) radical scavenging capacity and ferric reducing antioxidant power (FRAP) than Tib [6]. What is more, different geographical locations have a great impact on the active ingredients of the sea buckthorn berry [12]. The Qinghai–Tibet Plateau is an important area for the distribution, cultivation and utilization of sea buckthorn. Rha and Tib are the representative populations in this area. Their special geographical environment creates unique conditions for the accumulation of their active ingredients [13]. Although there have been reports regarding comparison of the phenolic and flavonoid contents as well as the antioxidant activities of the ethanol extracts of Rha and Tib, there is still limited literature available concerning comparison of the of soluble sugar, organic acid, free phenolic, bound phenolic, V_C_, V_E_ and carotenoid content, in addition to the cellular antioxidant activity of Rha and Tib berries from the Qinghai–Tibet Plateau.

The objectives of the study were to: (1) measure the types and contents of soluble sugars and organic acids; (2) analyze the types and contents of phenolics and flavonoids; (3) examine the types and contents of V_C_ and V_E_; (4) determine the types and contents of carotenoids; (5) evaluate in vitro and Caco-2 cellular antioxidant activities.

## 2. Materials and Methods

### 2.1. Materials

Rha and Tib berries were collected from Luqu County, Gannan Tibetan Autonomous Prefecture, China, in September 2020 (local latitude 34°90′ N, longitude 102°92′ E, approximately 3114 m above sea level). Fruit branches measuring approximately 30 cm were cut from the plants, put into packaging boxes (2 kg/box) and transported to the laboratory.

The human colorectal adenocarcinoma (Caco-2) cell line was purchased from Shanghai Bioleaf Biotech Co., Ltd. (Shanghai, China).

### 2.2. Biochemical Sampling

The berries of Rha and Tib were frozen with liquid nitrogen, the branches were shaken to shake the fruits off and the frozen fruits were quickly ground into a powder using a mechanical grinder (A11 basic, IKA-Werke GmbH & Co.KG, Staufen, Germany), and stored at −80 °C.

### 2.3. Determination of Soluble Sugar Content

The soluble sugar content was determined by referring to the method of Petreikov et al. [14] with some adjustments. Frozen powder (2.0 g) was extracted using 3 mL acetonitrile in a water bath at 60 °C (10 min), then centrifuged (8000× *g*, 20 min). The extraction was repeated once and centrifuged, and the supernatant was collected and purified through a 0.22 μm filter. The analysis was performed using a high-performance liquid chromatograph (1100, Agilent, Santa Clara, CA, USA) equipped with a differential refractive index detector from the same manufacturer. The separation was performed on a NH_2_ amino (4.6 × 250 mm, 5 μm) column (Zorbax, Agilent). Acetonitrile–water (75/25, *v*/*v*) at a 1 mL/min flow rate was used as the mobile phase at 30 °C. The standard calibration curve equations for glucose, fructose and sucrose were y = 203,913x − 9054.3 (R^2^ = 0.9992), y = 178,967x + 18,820 (R^2^ = 0.9992) and y = 204,997x − 8477.2 (R^2^ = 0.9993), respectively. The linear range of the calibration concentrations were 0.625, 1.25, 2.5, 5 and 10 mg/mL. Total sugars were obtained by summing the concentrations of the individual components. Soluble sugar content was expressed as mg/g FW (fresh weight).

### 2.4. Determination of Organic Acid Content

Organic acid content was determined by referring to the method of Coelho et al. [15]. Frozen powder (5.0 g) was extracted using 25 mL ultrapure water, and then centrifuged (8000× *g*, 15 min, 4 °C) and purified through a 0.22 μm filter. A Waters ACQUITYArc system (Waters, Milford, MA, USA) equipped with a 2998 PDA photodiode matrix detector was used for the determination, and all separations were performed on a Hi-PiexH (300 × 7.7 mm) column (Agilent). The samples were analyzed with mobile phase 0.2 mM sodium dihydrogen phosphate (pH 2.7) at 210 nm and a flow rate 0.5 mL/min. The standard calibration curve equations were as follows: quinic acid: y = 578.57x + 6175.5 (R^2^ = 1); malic acid: y = 1871.7x + 20,660 (R^2^ = 0.9993); tartaric acid: y = 4540.1x − 13,373 (R^2^ = 0.9997); citric acid: y = 1550.3x − 7312.6 (R^2^ = 0.9991); and succinic acid: y = 153,773x − 225,757 (R^2^ = 0.9995). The linear range of the calibration concentrations was 30, 35, 40, 45 and 50 μg/mL. Total acids were obtained by summing the concentrations of the individual components. Organic acid content was expressed as mg/g FW.

### 2.5. Determination of Phenolic and Flavonoid Content

Phenolic content was determined by referring to the method of Guo et al. [11]. Specifically, frozen powder (20 g) was repeatedly extracted with 50 mL 80% (*w*/*v*) acetone for 10 min, and the supernatant was collected after centrifugation (8000× *g*, 15 min, 4 °C). The residue was re-extracted four times using the same procedures, and all supernatants were combined and evaporated at 45 °C using a rotary evaporator (RE-52A, Shanghai Yarong Biochemical Instrument Co., Ltd., Shanghai, China); the free phenolic extracts obtained were dissolved in ultrapure water (final volume, 20 mL) for determination. The residue from the extraction of free phenolics was shaken at room temperature and digested with NaOH (4 mol/L, 30 mL) for 1 h. The mixture was acidified to pH 2.0 with concentrated hydrochloric acid and extracted four times with ethyl acetate (30 mL), and the supernatant was obtained by centrifugation. Subsequently, the ethyl acetate fractions were pooled and evaporated at 45 °C using a rotary evaporator (RE-52A, Shanghai Yarong Biochemical Instrument Co., Ltd.) to dryness, before being reconstituted in 20 mL of water and then stored at −80 °C until use. The phenolic and flavonoid results were mg GAE/100 g FW (gallic acid) and mg RE/100 g FW (rutin), respectively [6]. Total phenolics and total flavonoids were obtained by summing the concentrations of the individual components.

A Waters ACQUITYArc system (Waters) equipped with a 2998 PDA photodiode matrix detector was used for the determination, and all separations were performed on a ZORBAXSB-C18 (250 × 4.6 mm, 5 μm) column (Agilent). The samples were eluted with a gradient system consisting of solvent A (0.1% formic acid in water) and solvent B (acetonitrile), with a flow rate of 0.8 mL/min. The column temperature was maintained at 28 °C and the injection volume was 5 µL. The gradient elution program that followed was 0 min (10% B), 0–3.5 min (10–13% B), 3.5–4 min (13–15% B), 4–7.5 min (15–18% B), 7.5–8.3 min (18–20% B), 8.3–8.5 min (20–25% B), 8.5–9 min (25–30% B), 9–11 min (30–30% B), 9–11 min (30–30% B). 9–11 min (30–35% B), 11–12 min (35–40% B), 12–13 min (40–42% B), 13–15.5 min (42–44% B), 15.5–15.8 min (44–50% B), 15.8–16.1 min (50–55% B), 16.1–17 min (55–60% B), 17–18 min (60–65% B), 18–21 min (65–70% B), 21–25 min (70–90% B), 25–30 min (90–10% B) and 30–37 min (90–10% B). The phenolic compounds were identified via comparison with the retention time of the standard using PDA detection at 280 nm and 360 nm, respectively, and individual phenolic content was estimated on the basis of the peak area and the calibration curves of the corresponding standards. Phenolic content was expressed as mg/g FW.

### 2.6. Determination of V_C_ and V_E_ Content

V_C_ content was determined by referring to the method of Sytařová et al. [16] with a small adjustment. Frozen powder (0.1 g) was extracted using 1 mL of 1% (*w*/*v*) oxalic acid; the supernatant was harvested after centrifugation (8000× *g*, 10 min, 4 °C) and was fixed with 1% oxalic acid to 1 mL and purified through a 0.22 µm nylon membrane. A Kromasil C18 (250 × 4.6 mm, 5 μm) column (Akzo Nobel, Sweden) was used for HPLC (Rigol L3000, Beijing Puyuan Jingyi Technology Co., Ltd., China) analysis. HPLC conditions were as follows: mobile phases A was 0.1% oxalic acid and mobile phases B was methanol in the ratio of 95/5 (*v*/*v*). The flow rate was 0.8 mL/min, column temperature was 25 °C and the injection volume was 5 µL. The UV detection wavelength was 245 nm. V_C_ was characterized by retention time with standard compounds and quantified by peak area and calibration curve. The concentration of V_C_ in the sample was calculated with a standard curve. V_C_ content was expressed as mg/100 g FW.

V_E_ content was determined by referring to the method of Tkacz et al. [17]. Frozen powder (0.1 g) was extracted using anhydrous ethanol (2 mL, containing 0.1% butylated hydroxytoluene (BHT)) and 40 μL 50% KOH, and was heated at 85 °C for 5 min. Then, 1 mL precooled ultrapure water and 1 mL n-hexane were added, and the solution was mixed well. The supernatant (n-hexane) was harvested after centrifugation (2700× *g*, 10 min, 4 °C). N-hexane was then added to the centrifuged residue, and the supernatant was obtained by centrifugation. The process was repeated three times. Finally, all supernatants were combined and dried under a nitrogen stream; the residue was dissolved in 200 μL methanol and purified by passing through a 0.45 µm organic membrane. A YMC C30 (150 × 4.6 mm, 3 μm) column (YMC-Europe GmbH, Wesel, Germany) was used for ultrahigh-performance liquid chromatography (Ultimate 3000 DGLC, Thermo Fisher Scientific, Waltham, MA, USA) equipped with a fluorescence detector from the same manufacturer for analysis. The HPLC conditions were as follows: ultrapure water (A) and acetonitrile (B) as the mobile phases; gradient elution at 0–15 min, 30% A and 70% B; at 15–25 min, 80% A and 20% B; at 25–30 min, 30% A and 70% B; and at 30 min, 100% A and 0% B. The flow rate was 1 mL/min, column temperature was 40 °C and the injection volume was 2 µL. The fluorescence detector was set at 294 nm for excitation and 328 nm for emission. V_E_ was characterized by retention time with standard compounds (*γ*-V_E_, *α*-V_E_, *δ*-V_E_), and quantified by peak area and calibration curve. The concentration of each tocopherol in the sample was calculated with a standard curve. V_E_ content was expressed as mg/100 g FW.

### 2.7. Determination of Carotenoid Content

Carotenoid content was determined by referring to the method of Meléndez-Martínez et al. [18]. Frozen powder (0.1 g) was extracted using 1 mL of the extraction mixture (methanol/acetone/hexane, 25/25/50, *v*/*v*/*v*, containing 0.1% BHT) for 30 min in the dark; the supernatant was harvested after centrifugation (2500× *g*, 10 min, 4 °C). The supernatant was mixed with 10% ethanol and potassium hydroxide for 1 h, and washed twice with ultrapure water (saponification), which was the carotenoid extract. The extract was dried with a centrifugal vacuum concentrator (ZLS-1, Hunan Hercy Instrument Equipment Co., Ltd., Changsha, China) and dissolved in 0.2 mL of the acetone:methanol (1:2, *v*/*v*) mixture, and then purified by passing it through a 0.45 µm filter. A YMC C30 (150 × 4.6 mm, 3 μm) column (YMC-Europe GmbH) was used for ultrahigh-performance liquid chromatography (Ultimate 3000 DGLC, Thermo Fisher Scientific) with the mobile phase comprising methanol/methyl-*tert*-butyl ether (MTBE)/water (81/15/4, *v*/*v*/*v*) (A)-methanol (B) at a 450 nm wavelength. The elution was performed as described below: at 0–15 min, 100% A and 0% B; at 15–25 min, 39% A and 61% B; at 25–30 min, 100% A and 0% B; and at 30 min, 100% A and 0% B. The injection volume was 2 µL, flow rate was 1.0 mL/min and column temperature was 40 °C. The total carotenoid was obtained by summing the concentrations of the individual components. Carotenoid content was expressed as mg/100 g FW.

### 2.8. In Vitro Antioxidant Activity Assays

Antioxidant substances were extracted from the frozen powder of the berries (0.1 g) using ultrapure water (1 mL); the supernatant was harvested after centrifugation (8000× *g*, 15 min, 4 °C) for determination.

The DPPH, 2,2′-azino-bis (3-ethylbenzothiazoline-6-sulphonic acid (ABTS) and superoxide anion (O_2_^•−^) radical scavenging capacity were analyzed according to the methods of Brand-Williams et al. [19], Bao et al. [20] and Hsu et al. [21]. FRAP was conducted as referred to the method of Benzie et al. [22]. The antioxidant activities were expressed as mg TE/g FW, based on the Trolox (Sigma Aldrich, MO, USA) calibration curves.

### 2.9. Cell Cytotoxicity Assay

Caco-2 cells were cultured in advanced DMEM supplemented with 10% endotoxin-free, heat-inactivated fetal bovine serum (FBS), 1% L-glutamine, 1% penicillin (10,000 U/mL) and 1% streptomycin (10,000 μg/mL). Cells were cultured in a humidified atmosphere at 37 °C in 5% CO_2_ [23].

Cytotoxicity was measured using a CCK-8 agent. In the presence of electronically coupled reagents, WST-8 (2-(2-methoxy-4-nitrophenyl)-3-(4-nitrophenyl)-5-(2,4-disulfobenzene)-2H-tetrazolium monosodium salt) was reduced by dehydrogenase in the mitochondria to produce a highly water-soluble orange–yellow formazan product, whose color depth was inversely proportional to cytotoxicity. Frozen powder (10 mg) was dissolved in a serum-free cell culture medium (1 mL) and evenly mixed. After sterile filtration, the dilution concentrations were 0.15625, 0.3125, 0.625, 1.25, 2.5, 5 and 10 mg/mL. According to the Cell Counting Kit 8 (CCK-8, Medchem Express, Princeton, NJ, USA) method, Caco-2 cells (80 μL, 2 × 10^4^ cells/well) were sucked into each well of a 96-well plate and incubated at 37 °C and 5% CO_2_ for 24 h. Then, 20 μL solutions of various concentrations of sea buckthorn solution were added and placed under the same conditions for 24 h, with 20 μL of the serum-free cell culture medium used as the control group. Thereafter, the cell culture was removed and the wells were washed twice with DMEM; then, 10 μL of CCK-8 solution was added to each well and incubated for 4 h. The absorbance value was measured at 450 nm and cell viability was expressed as: cell viability (%) = (A_1_/A_0_) × 100%, where A_0_ represents the absorbance value of the control cells and A_1_ represents the absorbance value of the treated cells.

### 2.10. Cellular Antioxidant Activity (CAA) Assay

CAA was measured following the method of Kellett et al. [23]. CAA was determined with the 10 mg/mL sample solution detailed in Section 2.9 above. The standard used was quercetin. Specifically, 2 × 10^4^ cells/well Caco-2 cells were planted in a 96-well plate and incubated at 37 °C and 5% CO_2_ for 24 h; the medium was removed and cells were washed once with 100 μL PBS. Then, 100 µL of various concentrations of quercetin or 10 mg/mL sample were dissolved in the treatment medium containing 2′,7′-dichlorofluorescin diacetate (DCFH-DA) (25 μmol/L) and they were added in triplicate wells and incubated for 1 h. For the ‘PBS wash’ protocol, cells were washed with PBS; for the ‘no PBS wash’ protocol, cells were not washed prior to adding ABAP. The purpose of washing with PBS was to remove the interference of extracellular antioxidants. Finally, 2,2′-azobis (2-amidinopropane) dihydrochloride (ABAP) (100 μL, 600 μM) was added into each well, the purpose of ABAP treatment of cells is that ABAP disperses in the cells and spontaneously decomposes to form peroxyl radicals, which attack the cell membrane to generate more free radicals or reactive oxygen species. The fluorescence value for 1 h was recorded every 10 min with the emission wavelength at 538 nm and excitation at 485 nm. The CAA value was expressed as µmol QE/100 g FW.

### 2.11. Data Statistics

All analyses within an experiment were conducted using three biological replicates. Excel 2021 (Microsoft, Redmond, Washington, DC, USA)was used to calculate the mean and standard error (±SE). OriginLab OriginPro 8.5 (Northampton, MA, USA) was used for drawing. Duncan’s multiple difference significance analysis at a 5% level was performed using IBM SPSS Statistics 24.0 (IBM, Armonk, NY, USA) (*p* < 0.05).

## 3. Results and Discussion

### 3.1. The Content of Soluble Sugars in Rha and Tib Berries

**Figure 1:**
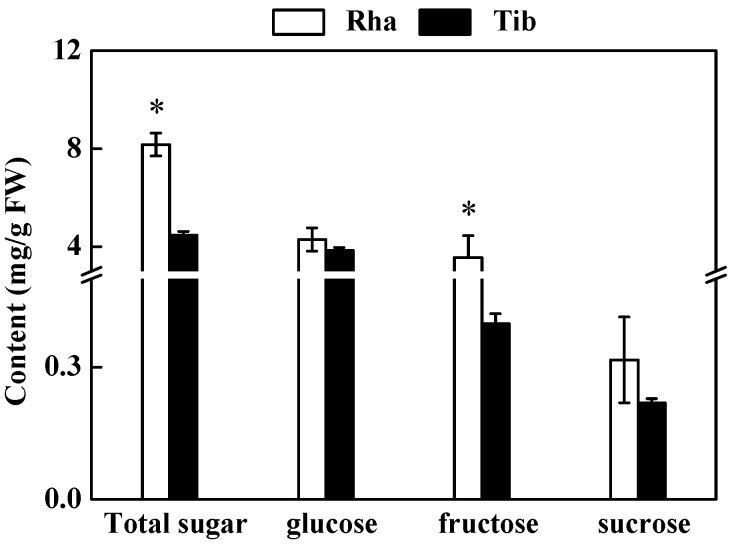
The content of soluble sugars in Rha and Tib berries. Vertical bars represent standard error of the mean (±SE), and the “*” indicate significant differences at *p* < 0.05.

The total sugar and fructose content in Rha were significantly higher than that in Tib, while there was no significant difference between the two sea buckthorn berries in terms of glucose or sucrose (Figure 1). Glucose and fructose were the main soluble sugars, accounting for over 90% of the total sugar content; the results of this study are in line with a previous report [24]. The soluble sugar content in our study was lower than the published value of 1.34–2.87 g/100 g for the six sea buckthorn cultivars commonly grown in Poland [17]. These differences may be caused by genotype, the growing environment, extraction methods and the harvest time [25]. In our research, Rha and Tib were collected at the same time in the same place in the Qinghai–Tibet Plateau area; then, they were extracted and analyzed using the same methods, so that environmental factors could be eliminated. Therefore, the difference in soluble sugar content between the two sea buckthorn berries was mainly due to the difference in genotype.

### 3.2. The Content of Organic Acids in Rha and Tib Berries

**Figure 2:**
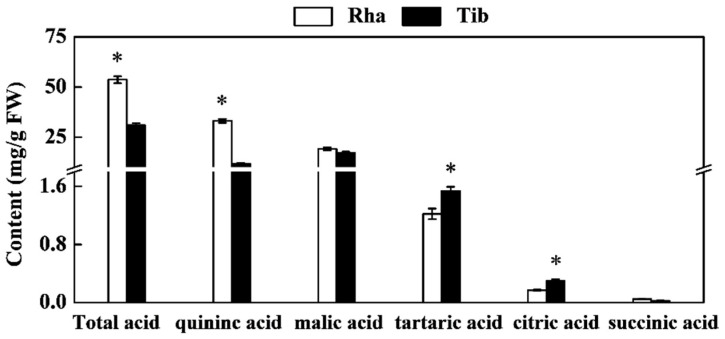
The content of organic acids in Rha and Tib berries. Vertical bars represent standard error of the mean (±SE), and the “*” indicate significant differences at *p* < 0.05.

Compared with Tib, Rha had a higher total acid and quinic acid content, but lower tartaric acid and citric acid content. There was no significant difference between the two sea buckthorn berries in terms of malic acid and succinic acid (Figure 2). Quinic acid and malic acid were the main organic acids, accounting for over 90% of the total acid content. The organic acid content of Rha in this study was higher than the published value of 0.96–4.22 g/100 g from the six sea buckthorn cultivars commonly grown in Poland [17], the biosynthesis and accumulation of sea buckthorn berries are influenced by intrinsic factors, such as genotype and developmental stage, as well as by external factors, such as light, temperature and precipitation. Furthermore, citric acid, succinic acid and malic acid are synthesized by the tricarboxylic acid cycle [26]; the higher citric acid content of Tib may be related to its strong tricarboxylic acid cycle activity. Quinic acid is synthesized in a branched chain of the shikimic acid pathway through the reversible reduction of 3-dehydroquinic acid by quinic acid dehydrogenase [27]; the higher quinic acid content of Rha may be related to its higher quinic acid dehydrogenase activity.

### 3.3. The Content of Phenolics, Flavonoids and Twelve Phenolics in Rha and Tib Berries

**Figure 3:**
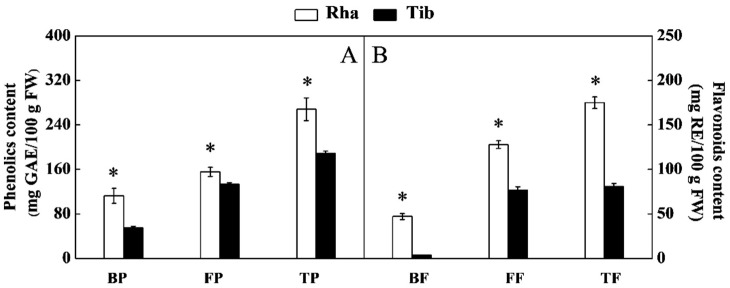
The content of phenolics (**A**) and flavonoids (**B**) in Rha and Tib berries. Vertical bars represent standard error of the mean (±SE), and the “*” indicate significant differences at *p* < 0.05. BP, bound phenolics; FP, free phenolics; TP, total phenolics; BF, bound flavonoids; FF, free flavonoids; TF, total flavonoids. (**A**) The content of phenolics in Rha and Tib berries; (**B**) The content of flavonoids in Rha and Tib berries.

Phenolic acids and flavonoids were the main phenolic substances in sea buckthorn, which are usually present as free and bound fractions [28]. The total phenolic content of Rha was 268.096 mg GAE/100 g, which was 42% higher than that in Tib. The free phenolic and bound phenolic contents of Rha were 16% and 103% higher than that in Tib, respectively (Figure 3A). The total flavonoid, free flavonoid and bound flavonoid contents of Rha were 117%, 66% and 1154%, respectively, which were much higher than those in Tib (Figure 3B). The free fraction content was much higher compared to the bound fractions, which is in alignment with previous data [11]. The above results showed that the total (free and bound) phenolic and the total (free and bound) flavonoid contents of Rha were obviously higher than those in Tib, which was in line with the results of Wei et al. [6], who reported that the total phenolic and total flavonoid contents of ethanolic extracts from Rha were significantly higher than those of Tib. This further indicates that genotype was the main reason for the difference in phenolic content.

In total, twelve phenolic compounds (Table 1) were divided into four categories. Benzoic acid derivatives (gallic acid, protocatechuic acid), flavanols (catechin, epicatechin), flavonols (isorhamnetin, kaempferol, myricetin, quercetin, naringenin) and phenylpropanoids (ferulic acid, chlorogenic acid, *p*-coumaric acid) were found in Rha and Tib berries. Among these, flavonols were dominant with 0.2069 ± 0.0375 mg/g FW, followed by flavanols, phenylpropanoids and benzoic acid derivatives (0.1482 ± 0.0098 mg/g FW, 0.1155 ± 0.0031 mg/g FW and 0.0180 ± 0.0009 mg/g FW, respectively) in Rha. Among the phenolics analyzed, the content of catechin in Rha was the highest (0.1345 ± 0.0085 mg/g FW), followed by isorhamnetin, quercetin and chlorogenic acid (0.1077 ± 0.0142 mg/g FW, 0.0662 ± 0.0115 mg/g FW and 0.0618 ± 0.0007 mg/g FW, respectively) in Rha. Tib showed the same trend as Rha. The gallic acid and protocatechuic acid content was lower than the results reported for four subspecies of sea buckthorn (*H. rhamnoides* L. subsp. *sinensis*, *H. rhamnoides* L. subsp. *yunnanensis*, *H. rhamnoides* L. subsp. *mongolica*, *H. rhamnoides* L. subsp. *turkestanica*) from China. The contents of catechin, isorhamnetin and quercetin were in alignment with the results reported for four subspecies of sea buckthorn from China [11]. On the whole, flavonoids accounted for approximately 80% of the phenolic content, which was in alignment with the data described by Guo et al. [11], indicating that flavonoids were the main phenolic substances. The type and concentration of phenolics have been reported to be influenced by genotype and growing conditions [29]. The genotype may be the primary cause of the variance between the two sea buckthorn berries, because they were both picked from the same location. As important secondary metabolites of plants, phenolics can only be synthesized via phenylpropane metabolism [30]. The phenylalanine content of the initial substrate of phenylpropane metabolism is an important factor responsible for the differences in phenolic content among sea buckthorn [25,30]. The activities of phenylalanine deaminase, cinnamic acid-4-hydroxylase and 4-coumaroyl-CoA ligase led to the difference in phenolic acid levels, and the activities of chalcone synthase and flavonoid-3′,5′-hydroxylase led to the difference in flavonoid levels among sea buckthorn berries [25]. Furthermore, free phenolics do not interact either physically or chemically with other molecules. In contrast, bound phenolics that are bound to cell wall polysaccharides or proteins form insoluble stable complexes [28]. Thereby, we speculate that the higher total phenolics and total flavonoids of Rha may be related to its higher phenylalanine content and stronger phenylpropane metabolic activity. Rha has high levels of bound phenolics and bound flavonoids, which may be related to the richness in cell wall polysaccharides.

### 3.4. The Content of V_C_ and V_E_ in Rha and Tib Berries

**Figure 4:**
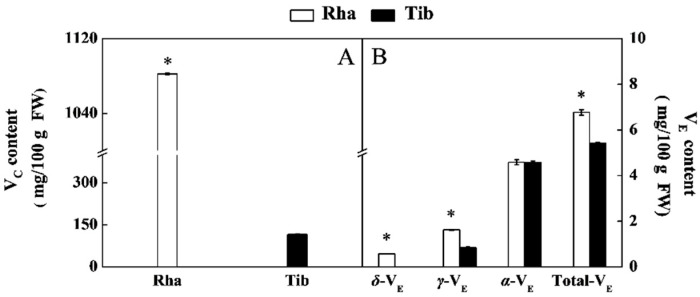
The content of V_C_ (**A**) and V_E_ (**B**) in Rha and Tib berries. Vertical bars represent standard error of the mean (±SE), and the “*” indicate significant differences at *p* < 0.05. (**A**) The content of V_C_ in Rha and Tib berries; (**B**) The content of V_E_ in Rha and Tib berries.

As another abundant component in the sea buckthorn berry, V_C_ has a very high antioxidant effect [7]. The V_C_ content of Rha was 1080.30 mg/100 g, which was 836% higher than that of Tib (Figure 4A). The V_C_ content of Rha was significantly higher than the published value of 1.84–3.46 g/kg from the five sea buckthorn cultivars from the Hexi corridor in China, while the content of Tib was slightly lower than the reported results [31]. These differences may be due to the difference in genotype. Furthermore, V_C_ in sea buckthorn is synthesized through five pathways: the L-galactose pathway, L-gulose pathway, D-galacturonate pathway, myo-inositol pathway and uronic acid pathway. Of these, the L-galactose pathway is the most crucial. In addition, the activities of GDP-L-galactose phosphorylase, GDP-D-mannose-3′,5′-epimerase and L-galactose dehydrogenase have led to differences in V_C_ levels among different sea buckthorn varieties [25]. Consequently, we surmised that the higher V_C_ content of Rha may be related to its stronger L-galactose metabolic activity.

In addition to its high vitamin C content, sea buckthorn berries are relatively abundant in tocopherol [4]. Total V_E_ and *γ*-V_E_ contents of Rha were 25% and 38% higher than that in Tib, respectively. Of these, *α*-V_E_ was the major V_E_ in Rha and Tib. Notably, Rha contained traces of *δ*-V_E_, but this was not detected in Tib (Figure 4B). The above results indicate that total V_E_, *δ*-V_E_ and *γ*-V_E_ contents of Rha were remarkably higher than those in Tib. The total V_E_ content in this study was lower than the published value of 27.12–34.27 g/100 g from the six sea buckthorn cultivars commonly grown in Poland [17]; these differences may be due to the difference in genotype. Furthermore, the V_E_ of the sea buckthorn berry is synthesized via the shikimate pathway and the nonmevalonate pathway [32]. The activities of homogentisate phytyltransferase, tocopherol cyclase and 2-methyl-6-phytylbenzoquinol methyltransferase led to the differences in V_E_ levels among sea buckthorn berries [25]. Hence, we speculate that the higher V_E_ content of Rha may be related to the stronger shikimate pathway and the metabolic activity of the nonmevalonate pathway.

### 3.5. The Content of Carotenoids in Rha and Tib Berries

**Figure 5:**
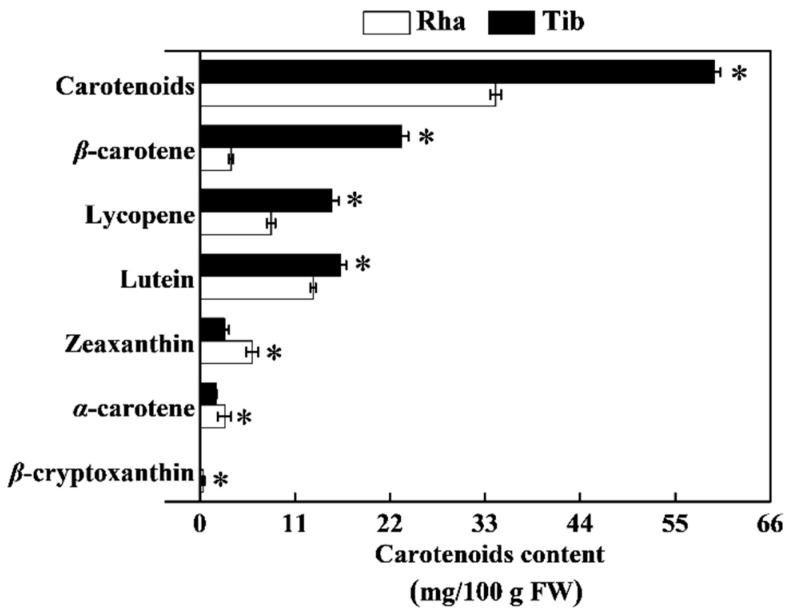
The content of carotenoids in Rha and Tib berries. Vertical bars represent standard error of the mean (±SE), and the “*” indicate significant differences at *p* < 0.05.

The total carotenoid content of Tib was 59.56 mg/100 g, which was 73% higher than that of Rha. Notably, Rha contained traces of *β*-cryptoxanthin, which was not detected in Tib. The contents of *α*-carotene and zeaxanthin in Rha were 53% and 113% higher than those in Tib, while the lutein, *β*-carotene and lycopene contents in Tib were 23%, 547% and 84% higher than those in Rha, respectively, with *β*-carotene and lycopene being the main carotenoids (Figure 5). The carotenoid content of sea buckthorn berries in this study was slightly lower than the values of 53.1–96.7 g/kg reported from six Romanian sea buckthorn (*Hippophae rhamnoides* L.) varieties [33]. The difference may be due to the difference in genotype. It has also been reported that the carotenoid content of five varieties of sea buckthorn from Poland (Botaniczeskaja Ljubitelskaja, GoldenRain, Prozracznaja, Maryna, Luczystaja) was 671–1418 mg/kg. Among the nine types of carotenoids identified, all-*trans*-*β*-carotene and *β*-cryptoxanthin were dominant [10]. In addition, carotenoids of the sea buckthorn berry are mainly synthesized via the methylerythritol phosphate pathway. The activities of phytoene synthase and 9-*cis*-epoxycarotenoid dioxygenase led to the difference in carotenoid levels among sea buckthorn berries [30]. Thereby, we speculate that the higher carotenoid content of Tib may be related to its active methylerythritol phosphate pathway.

### 3.6. In Vitro Antioxidant Activity of Rha and Tib Berries

**Figure 6:**
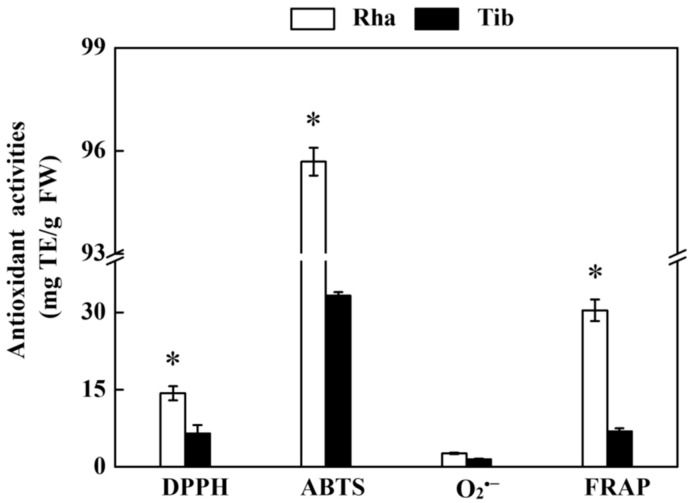
In vitro antioxidant activities of Rha and Tib berries. Vertical bars represent standard error of the mean (±SE), and the “*” indicate significant differences at *p* < 0.05.

DPPH, ABTS, O_2_^•−^ free radical scavenging activity and FRAP are common indices to evaluate antioxidant capacity. Both Rha and Tib showed a good in vitro antioxidant activity, with the strongest scavenging activity being exhibited by ABTS, at 95.68 and 33.23 mg TE/g FW, followed by FRAP, DPPH and O_2_^•−^, in that order. The DPPH, ABTS and FRAP of Rha were 120%, 187% and 341% higher than those in Tib, respectively (Figure 6), which is in alignment with previous data [6]. Moreover, the antioxidant mechanism differs among different antioxidant substances [34]. According to studies, the redox characteristics of phenolics play a major role in the antioxidant activity of sea buckthorn [35,36]. Phenolics react directly with free radicals as hydrogen donors [4]. V_C_ can transfer electrons to free radicals [37]. Tocopherols quench reactive oxygen species by scavenging lipid peroxidation free radicals. Carotenoids form low-reactive free radical products by reacting with free radicals [38]. Furthermore, polyphenolics appear to be the most effective antioxidants, as indicated by the extremely strong correlations between total phenolics and DPPH in berries (r = 0.8904) [39]. It is reported that catechin and *p*-coumaric acid in the sea buckthorn berry showed a positive correlation with DPPH [17], the synergistic effect of individual phenolic compounds may lead to the enhancement of in vitro antioxidant activities. Isorhamnetin, quercetin and kaempferol were found to be the most potent flavonol glycosides in term of free radical scavenging activity by Chen et al. [40]; V_C_ affected the DPPH antioxidant activity of the sea buckthorn berry (r = 0.8247) and V_E_ was weakly correlated with DPPH. Correspondingly, *β*-carotene and lycopene also scavenge ABTS free radicals [17]. These results illustrate that the in vitro antioxidant activities of Rha were notably better than that in Tib, which may be due to the important role played by phenolics, flavonoids, V_C_ and V_E_. 

### 3.7. Cell Cytotoxicity and CAA of Rha and Tib Berries

**Figure 7:**
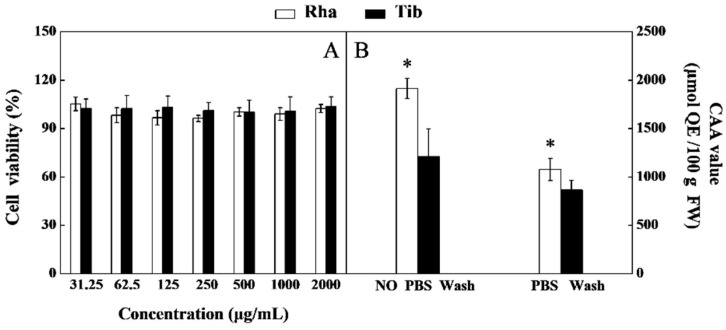
Cell viability (**A**) and CAA (**B**) of Rha and Tib berries. Vertical bars represent standard error of the mean (±SE), and the “*” indicate significant differences at *p* < 0.05. (**A**) Cell viability of Rha and Tib berries; (**B**) CAA of Rha and Tib berries.

Caco-2 cells are morphologically and structurally similar to small intestinal epithelial cells, suggesting that they may be a superior indicator of antioxidant activity in vivo [23]. The cytotoxicity of the test components to the cells must be evaluated before assessing their antioxidant activity. The results revealed that cell survival was higher than 96% when treated at concentrations of 31.25–2000 μg/mL (Figure 7A). It was shown that the sea buckthorn berry did not show toxicity to Caco-2 cells in this concentration range.

The CAA method is a reasonable method for assessing antioxidant activity based on cell culture models, reflecting the absorption and metabolism of antioxidants, and the results have high biological relevance. Additionally, this method includes a ‘PBS wash’ method and a ‘no PBS wash’ method to evaluate the degree of uptake and membrane association of antioxidants [23]. When the cells were washed without PBS, the CAA values of Rha and Tib were 1915.38 μmol QE/100 g FW and 1077.83 μmol QE/100 g FW, respectively. When they were washed with PBS, the CAA values of Rha and Tib were 1210.56 μmol QE/100 g FW and 866.20 μmol QE/100 g FW, respectively (Figure 7B). Regardless of whether the cells were washed with PBS or not, the CAA value of Rha was always higher than that of Tib; this trend agrees with the in vitro antioxidant activity (DPPH, ABTS, FRAP) assay results. It further demonstrated that the antioxidant activity of the sea buckthorn berry may be influenced by phenolics, flavonoids and V_C_. In general, the total contribution of isorhamnetin, quercetin and kaempferol in the CAA was around 20% [41], since the contents of isorhamnetin, quercetin and kaempferol in the Rha berry are higher than those in Tib. This may be an important reason for the higher CAA of Rha. Moreover, the CAA of sea buckthorn berries washed without PBS was higher than the CAA when the berries were washed with PBS; the results are in alignment with those reported for other fruits [42]. This phenomenon could be a result of some of the antioxidant components of sea buckthorn berries being loosely attached to the membrane so that they were removed when the berries were washed with PBS [42]. The above results suggest that the higher content of phenolics, flavonoids, and V_C_ in Rha is an important reason for its high CAA value.

## 4. Conclusions

The total sugar, fructose, total acid, quinic acid, total (free and bound) phenolic, and total (free and bound) flavonoid contents of Rha were considerably higher than that in Tib. Twelve phenolic compounds were analyzed, among which flavonols were dominant. Catechin, isorhamnetin, quercetin and chlorogenic acid were the main phenolic substances. The V_C_ and V_E_ (*γ*-V_E_, *δ*-V_E_) of Rha were remarkably higher than those of Tib. The total carotenoid, lutein, *β*-carotene and lycopene contents of Tib were notably higher than that in Rha. Furthermore, both Rha and Tib showed good in vitro and cellular antioxidant activity, and Rha significantly outperformed Tib in terms of scavenging DPPH, ABTS free radicals, FRAP and its antioxidant capacity against Caco-2 cells. Thus, Rha berries could be considered for special medical or functional food processing due to their strong antioxidant activity.

## Figures and Tables

**Table 1:** Phenolic compound content of Rha and Tib berries.

Phenolic Compounds (mg/g FW)	Free Phenolics	Bound Phenolics	Total Phenolics
Benzoic acid derivatives			
Gallic acid			
Rha	0.0081 ± 0.0004 ^a^ (51.59)	0.0076 ± 0.0003 ^a^ (48.41)	0.0157 ± 0.0007 ^a^
Tib	0.0091 ± 0.0004 ^a^ (62.32)	0.0054 ± 0.0002 ^a^ (37.68)	0.0146 ± 0.0006 ^a^
Protocatechuic acid			
Rha	0.0012 ± 0.0001 ^a^ (54.54)	0.0011 ± 0.0000 ^a^ (45.46)	0.0023 ± 0.0002 ^a^
Tib	0.0016 ± 0.0004 ^a^ (69.56)	0.0007 ± 0.0002 ^a^ (30.44)	0.0023 ± 0.0006 ^a^
Sum			
Rha	0.0093 ± 0.0005 ^b^ (51.66)	0.0087 ± 0.0003 ^a^ (48.34)	0.0180 ± 0.0009 ^a^
Tib	0.0107 ± 0.0008 ^a^ (63.31)	0.0061 ± 0.0004 ^b^ (36.69)	0.0169 ± 0.0012 ^b^
Flavanols			
Catechin			
Rha	0.1083 ± 0.0064 ^a^ (80.58)	0.0262 ± 0.0021 ^a^ (19.42)	0.1345 ± 0.0085 ^a^
Tib	0.0472 ± 0.0069 ^b^ (76.99)	0.0141 ± 0.0038 ^b^ (23.01)	0.0613 ± 0.0108 ^b^
Epicatechin			
Rha	0.0093 ± 0.0012 ^a^ (67.39)	0.0044 ± 0.0000 ^a^ (32.61)	0.0137 ± 0.0013 ^a^
Tib	0.0052 ± 0.0001 ^b^ (54.16)	0.0043 ± 0.0000 ^a^ (45.84)	0.0096 ± 0.0001 ^b^
Sum			
Rha	0.1176 ± 0.0076 ^a^ (79.35)	0.0306 ± 0.0021 ^a^ (20.65)	0.1482 ± 0.0098 ^a^
Tib	0.0524 ± 0.0070 ^b^ (73.90)	0.0184 ± 0.0038 ^b^ (26.10)	0.0709 ± 0.0109 ^b^
Flavonols			
Isorhamnetin			
Rha	0.0966 ± 0.0132 ^a^ (89.69)	0.0111 ± 0.0009 ^a^ (10.31)	0.1077 ± 0.0142 ^a^
Tib	0.0397 ± 0.0063 ^b^ (76.19)	0.0124 ± 0.0022 ^a^ (23.81)	0.0521 ± 0.0085 ^b^
Kaempferol			
Rha	0.0039 ± 0.0001 ^a^ (54.16)	0.0033 ± 0.0000 ^a^ (45.84)	0.0072 ± 0.0001 ^a^
Tib	0.0034 ± 0.0000 ^a^ (50.00)	0.0034 ± 0.0000 ^a^ (50.00)	0.0068 ± 0.0000 ^a^
Myricetin			
Rha	0.0197 ± 0.0012 ^a^ (94.71)	0.0011 ± 0.0000 ^a^ (5.29)	0.0208 ± 0.0012 ^a^
Tib	0.0095 ± 0.0006 ^b^ (85.58)	0.0015 ± 0.0002 ^a^ (14.42)	0.0111 ± 0.0009 ^b^
Quercetin			
Rha	0.0612 ± 0.0107 ^a^ (92.31)	0.0050 ± 0.0007 ^a^ (7.69)	0.0662 ± 0.0115 ^a^
Tib	0.0143 ± 0.0034 ^b^ (74.09)	0.0049 ± 0.0003 ^a^ (25.91)	0.0193 ± 0.0038 ^b^
Naringenin			
Rha	0.0024 ± 0.0000 ^a^ (48.97)	0.0025 ± 0.0000 ^a^ (51.03)	0.0049 ± 0.0000 ^a^
Tib	0.0025 ± 0.0000 ^a^ (50.00)	0.0025 ± 0.0000 ^a^ (50.00)	0.0050 ± 0.0000 ^a^
Sum			
Rha	0.1838 ± 0.0350 ^a^ (88.83)	0.0230 ± 0.0022 ^a^ (16.17)	0.2069 ± 0.0375 ^a^
Tib	0.0694 ± 0.0397 ^b^ (73.75)	0.0247 ± 0.0081 ^a^ (26.25)	0.0941 ± 0.0481 ^a^
Phenylpropanoids			
Ferulic acid			
Rha	0.0073 ± 0.0003 ^a^ (53.67)	0.0064 ± 0.0005 ^a^ (46.33)	0.0136 ± 0.0008 ^a^
Tib	0.0046 ± 0.0008 ^b^ (40.35)	0.0068 ± 0.0015 ^a^ (59.65)	0.0114 ± 0.0023 ^b^
Chlorogenic acid			
Rha	0.0615 ± 0.0031 ^a^ (99.51)	0.0003 ± 0.0001 ^a^ (0.49)	0.0618 ± 0.0007 ^a^
Tib	0.0286 ± 0.0031 ^b^ (98.96)	0.0003 ± 0.0000 ^a^ (1.04)	0.0289 ± 0.0031 ^b^
*P*-coumaric acid			
Rha	0.0329 ± 0.0009 ^a^ (80.04)	0.0073 ± 0.0007 ^a^ (19.96)	0.0402 ± 0.0016 ^a^
Tib	0.0079 ± 0.0015 ^b^ (48.76)	0.0082 ± 0.0012 ^a^ (51.24)	0.0162 ± 0.0028 ^b^
Sum			
Rha	0.1016 ± 0.0043 ^a^ (87.96)	0.0139 ± 0.0012 ^b^ (12.04)	0.1155 ± 0.0031 ^a^
Tib	0.0411 ± 0.0054 ^b^ (72.61)	0.0153 ± 0.0027 ^a^ (27.39)	0.0564 ± 0.0082 ^b^
Total			
Rha	0.4124 ± 0.0147 ^a^ (84.40)	0.0762 ± 0.0033 ^a^ (15.6)	0.4886 ± 0.0142 ^a^
Tib	0.1736 ± 0.0288 ^b^ (72.94)	0.0645 ± 0.0032 ^a^ (27.06)	0.2383 ± 0.0037 ^b^

Data are expressed as mean ± standard deviation (*n* = 3), different letters between two samples indicate statistically significant difference at *p* < 0.05 in the Duncan test. The values in parentheses indicate the proportion of compounds of the total amount (%).

## Data Availability

The data used to support the findings of this study can be made available by the corresponding author upon request.
